# Identification of *de novo* variants from parent-proband duos via long-read sequencing

**DOI:** 10.1101/2025.02.24.25322424

**Published:** 2025-02-26

**Authors:** Leandros Boukas, Emmanuèle C. Délot, Georgia Pitsava, Christine Lambert, Cairbre Fanslow, Primo Baybayan, Sami Belhadj, Bojan Losic, John Harting, Krista Bluske, Jonathan LoTempio, Huda Al-Kouatly, Rachid Karam, William Rowell, Changrui Xiao, Eric Vilain, Seth I. Berger

**Affiliations:** 1 Children’s National Hospital, Washington, DC; 2 UCI – GREGoR; 3 University of California, Irvine, CA; 4 PacBio, Menlo Park, CA; 5 Ambry Genetics, Aliso Viejo, CA; 6 Thomas Jefferson University, Philadelphia, PA; 7 The George Washington University, Washington, DC

## Abstract

While *de novo* variants cause many Mendelian disorders, their detection currently requires sequencing of the proband and both biological parents. This is not feasible when only one parent is available, a limitation for millions of families. We developed *duoNovo*, which identifies *de novo* variants from single parent-proband duos using long-read sequencing followed by haplotype reconstruction and detection of identical-by-descent haplotype blocks. We sequenced 40 trios and applied *duoNovo* to each of the 80 duos constructed by masking one parent, classifying over 20 million variants. We evaluated *duoNovo*’s performance against classifications obtained using the full trios (which included over 1,900 *de novo* variants), demonstrating very high precision and low error rate, and perfect accuracy among variants absent from gnomAD. *duoNovo* is freely available to the community as an R package, and may represent an example where long-read sequencing provides clear diagnostic benefit over short-read sequencing.

## Introduction

As genetic testing becomes standard of care for numerous clinical indications, it is crucial to consider social determinants that can lead to inequities in result interpretation and diagnostic outcomes. Diversity in family structure creates common scenarios where variant interpretation is hindered by the unavailability of samples from both biological parents. This is particularly relevant for *de novo* variants, which are present in affected probands but not (constitutively) present in the parents. *De novo* variants are recognized as underlying a substantial fraction of Mendelian disorders (Brunet et al., 2021; Ligt et al., 2012; Posey et al., 2017; Vissers et al., 2010; Wright et al., 2023; Yang et al., 2014). These disorders are typically highly penetrant, and as a result, the American College of Medical Genetics (ACMG) guidelines use a variant’s *de novo* status as a strong criterion for pathogenicity (Richards et al., 2015).

Currently, *de novo* status can only be determined through sequencing of the proband and both biological parents. However, various factors can prevent both parents from participating in genetic testing. US Census statistics indicate that there are approximately 10 million single-parent families in the United States alone (predominantly single-mother families). Family structures are further complicated by factors such as misattributed parentage, children conceived through sperm or egg donation, or parental death. Consequently, probands in these families face a higher risk of non-diagnostic results (Rehm et al., 2023; Richards et al., 2015; Wojcik et al., 2024). Given that ACMG guidelines advise against using VUS in treatment plan determinations, this can lead to missed opportunities to benefit from precision treatment and management options. Importantly, the magnitude of the reduction in diagnostic yield due to the unavailability of parental samples correlates with ancestry, indicating the potential for further exacerbation of existing health disparities (Wright et al., 2023).

Long-read sequencing (LRS) is emerging in the clinical setting, although higher cost combined with uncertainty regarding diagnostic benefit over short-read sequencing (SRS) currently limit its widespread adoption (Wojcik et al., 2023). To characterize the potential diagnostic benefit, initial investigations have largely focused on the ability of LRS to detect large/complex structural variants and other variants missed by SRS (Cohen et al., 2022; Mastrorosa et al., 2023; Merker et al., 2018; Miller et al., 2021; Mizuguchi et al., 2021; Negi et al., 2025; Xie et al., 2020); an additional studied benefit is the concurrent detection of epigenetic variation (Vollger et al., 2025). However, recent improvements in LRS technologies, such as the development of PacBio HiFi LRS, now allow one to obtain reads with very high basecall accuracy (Gustafson et al., 2024; Hiatt et al., 2024; Kolmogorov et al., 2023; Kucuk et al., 2023; Mastrorosa et al., 2023; Wenger et al., 2019), enabling the identification of small variants including single-nucleotide variants. One unique advantage of LRS is that it couples the identification of such variants with their reliable, read-backed phasing (Holt et al., 2024; Martin et al., 2016). This is especially important for potentially disease-causing variants evaluated for *de novo* status in the clinical diagnostic setting, which are very rare or even unique to an affected individual and thus not amenable to phasing methods that rely on information about haplotype frequencies in the population (Browning and Browning, 2011; Guo et al., 2024).

Here, we leverage these features of LRS to develop a method - which we call *duoNovo* - to identify *de novo* variants from duos (proband and one biological parent), with the potential to open more diagnostic opportunities for these families.

## Results

### *duoNovo*: overview of approach

**Figure 1.**
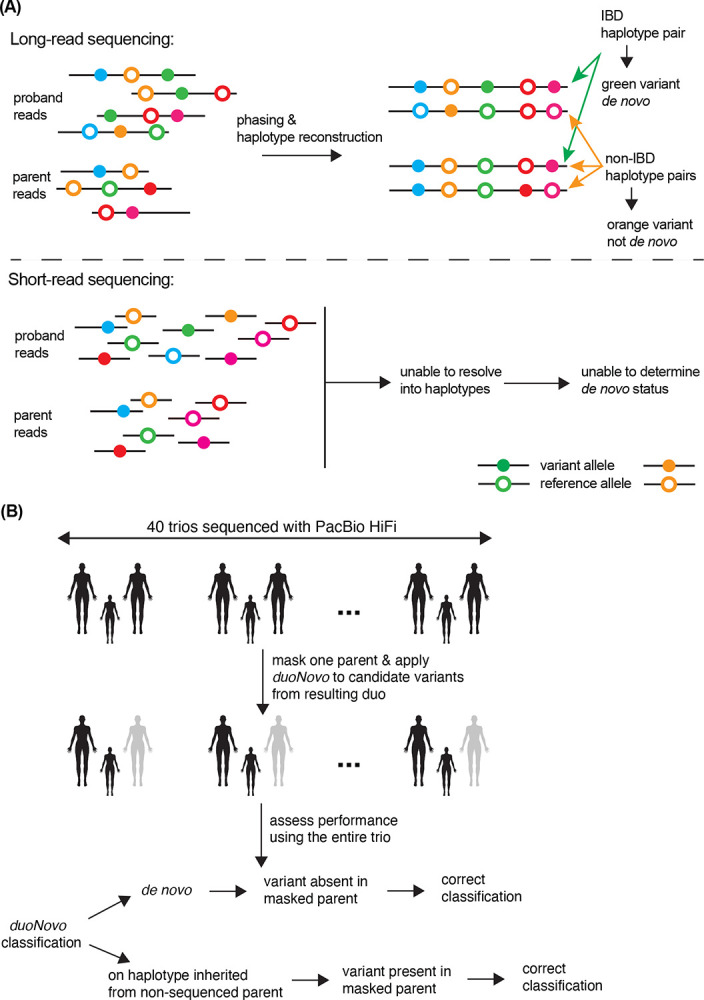
Detection of *de novo* variants from duos with *duoNovo*: conceptual basis and overview of strategy for performance testing. **(A)** The long reads produced by PacBio HiFi sequencing enable the read-backed phasing of variants and the subsequent reconstruction of haplotypes. *duoNovo* leverages this to identify pairs of proband-parent haplotypes that are identical-by-descent based on their sequence similarity. It then classifies candidate variants (heterozygous in the proband; absent in the parent) on such identical-by-descent haplotypes as *de novo* (orange variant in the cartoon figure; see also Methods). On the other hand, candidate variants on haplotypes not identical-by-descent with any of the two parent haplotypes (green variant in the cartoon figure) are inferred to have arisen on a haplotype inherited from the non-sequenced parent, in which case ascertaining their *de novo* status is not possible. This approach would not be feasible with short-read sequencing, as the short length of reads would preclude phasing and haplotype reconstruction. **(B)** We sequenced 40 trios with PacBio HiFi sequencing (Methods), and constructed father-proband and mother-proband duos by masking the mothers or the fathers, respectively. *duoNovo* was then applied to candidate variants from each duo, and the resulting variant classifications were evaluated based on the classifications that one would have obtained by having access to the entire trio.

*duoNovo* uses haplotype blocks consisting of phased single nucleotide variants across hundreds of kilobases in order to determine whether a candidate variant arose on the haplotype inherited from the sequenced parent – and is thus a *de novo* variant – or not (Methods). It achieves this by evaluating the sequence similarity between pairs of haplotype blocks. Each pair consists of a proband haplotype block and a parental haplotype block. If the proband haplotype block containing the variant being tested for *de novo* status exhibits perfect similarity (excluding the candidate variant) with one of the two parental haplotype blocks, then these haplotype blocks are inferred to be identical-by-descent, which in turn implies that the candidate variant is *de novo* (Figure 1A; Methods). If, on the other hand, it is the other proband haplotype (the one not containing the candidate variant) that exhibits perfect similarity with one of the two parental haplotype blocks, then *duoNovo* infers that the haplotype containing the candidate variant was inherited from the non-sequenced biological parent (Figure 1A). While in this latter case the candidate variant may still be *de novo*, this is not possible to determine from the available duo.

### *duoNovo* has high positive and negative predictive value

To assess the performance of *duoNovo*, we sequenced 40 trios using PacBio HiFi LRS to an average depth of ~ 33-fold across all individuals, obtaining approximately 17.7 kb-long reads on average (Methods).

**Figure 2.**
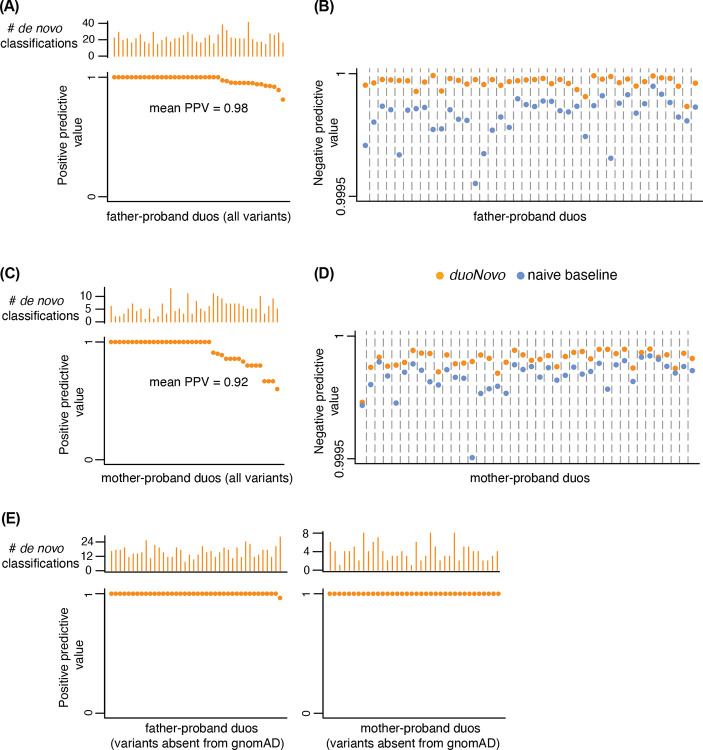
*duoNovo* has high positive and negative predictive value. **(A)** The positive predictive value of *duoNovo* (bottom panel y axis) and the number of variants classified as *de novo* (top panel y axis axis). Each point and its associated vertical bar above correspond to a father-proband duo. **(B)** The negative predictive value (y axis) of *duoNovo* (orange points) and of the naive baseline approach (blue points) for each father-proband duo (indexed on the x axis). The naive baseline approach consists of classifying every candidate variant as non-*de novo*. **(C)** and **(D)** Like **(A)** and **(B)**, but for mother-proband duos. Note that in **(A)** and **(C)**, duos are ordered on the x axis in decreasing positive predictive value, and thus the order is different between **(A)** and **(C)**. **(E)** Like **(A)** and **(C)**, but restricting to variants that are absent from gnomAD 4.1. In 4 mother-proband duos, there were no *de novo* classifications among variants absent from gnomAD, hence these duos are not visualized.

We started by applying *duoNovo* to each of the 40 father-proband duos, treating the mother as the non-sequenced parent (Figure 1B). We identified an average of 1,455,871 candidate variants across these duos (heterozygous phased variant call in the proband; homozygous reference call in the father). We subjected each candidate variant to strict quality control (QC; including sequencing depth, genotype quality, and regional quality) and tested each variant that passed our QC filters for *de novo* status (Methods). Across all 40 duos, an average of 24 variants (range 14–47) were classified as *de novo* (Figure 2A). Reassuringly, the number of *de novo* classifications positively correlates with father’s age (Supplemental Figure S1; Poisson regression *p* = 7.04 · 10^−9^). To evaluate the accuracy of the classifications, we examined the maternal sequence. For each variant classified as *de novo*, we determined that the classification is correct if the variant is absent in the mother (Figure 1B). We note that this a conservative assessment, as a variant can still be *de novo* even if it is present in the mother (for example, at recurrently mutated sites). We found that the average positive predictive value of *duoNovo* across duos is 98% (Figure 2A; range 81 – 100%). While the negative predictive value is expected to be high because *de novo* variants are extremely rare, we found that *duoNovo* always outperforms the naive baseline (calling every variant as non-*de novo*) (Figure 2B).

We next turned our attention to the mother-proband duos, this time treating the fathers as the non-sequenced parents. As expected given the elevated contribution of the paternal germline to the pool of *de novo* variants (Jónsson et al., 2017; Kong et al., 2012; Sasani et al., 2019), *duoNovo* always detected more *de novo* variants in father-proband duos compared to mother-proband duos (with the median ratio of paternally to maternally derived *de novo* classified variants being 4.2; Supplemental Figure S2). This can explain why, while the positive predictive value in mother-proband duos remains high, it is somewhat lower compared to father-proband duos (Figure 2C; average 92%; range 60–100%). As in father-proband duos, *duoNovo* always has higher negative predictive value compared to the naive baseline (Figure 2D).

In both father-proband and mother-proband duos, we observed no difference in the positive predictive value when the proband was of European vs non-European ancestry (Supplemental Figure S3; Methods)

### *duoNovo* has perfect accuracy when applied to candidate variants absent from gnomAD

In the previous section, we quantified the positive predictive value of *duoNovo* among all variants classified as *de novo*. In a realistic diagnostic setting, the potential pathogenicity of candidate variants evaluated for *de novo* status is supported by additional lines of evidence, an important one being their absence (or very low frequency) in control population databases. To evaluate the performance of *duoNovo* in such a setting, we focused on candidate *de novo* variants that are absent from gnomAD (Karczewski et al., 2020), and therefore have a higher prior probability of being *de novo* and pathogenic. *duoNovo* achieved perfect accuracy. Among 629 variants classified as *de novo* from father-proband duos and 142 variants classified as *de novo* from mother-proband duos, the positive predictive value is 100% in all but one of father-proband duos, and all mother-proband duos (Figure 2E).

### *duoNovo* very rarely classifies reference alleles as *de novo*

Our results so far focus on candidate alternative alleles (1|0 or 0|1 proband genotype; 0/0 parental genotype). As an additional check of *duoNovo*’s performance, we compared the percentage of candidate alternative alleles classified as *de novo* to the corresponding percentage for candidate reference alleles (1|0 or 0|1 proband genotype; 1/1 parental genotype). Collectively, alternative candidate alleles were ~ 97 times more likely to be classified as *de novo* compared to reference candidate alleles in father-proband duos, and ~ 11 times in mother-proband duos. In 36 out of the 40 father-proband duos and 30 out of the 40 mother-proband duos, no reference alleles were classified as *de novo* (Supplemental Figure S5). This further supports the accuracy of our *de novo* classifications, since the reference allele is common in the population and thus candidate reference alleles have a very low prior probability of being *de novo*.

### *duoNovo* has very low false positive rate

**Figure 3.**
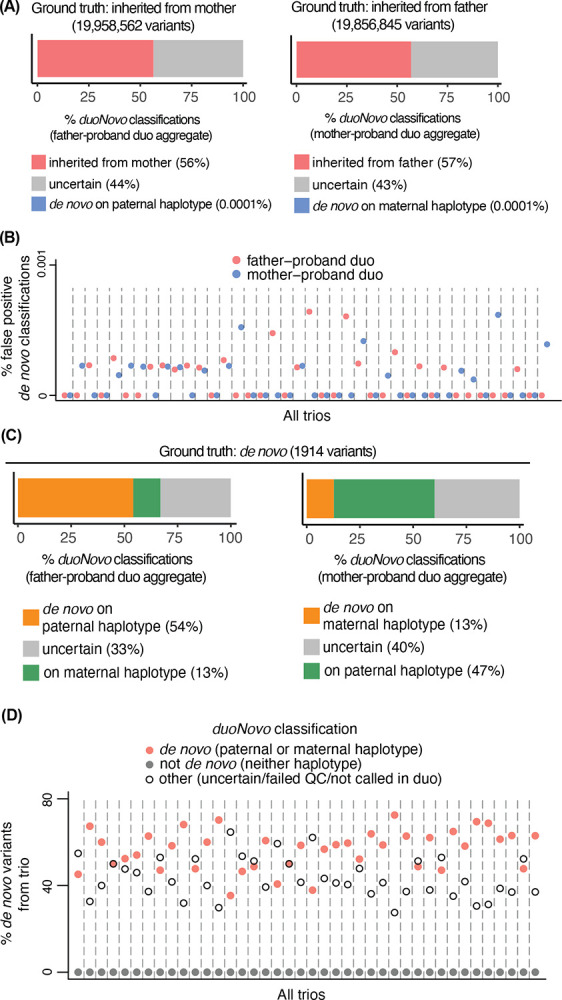
*duoNovo* has very low error rate. **(A)** The percentage of candidate variants present in the non-sequenced parent (and thus inferred to be non-*de novo*) that are classified by *duoNovo* as present on the haplotype inherited from the masked parent, *de novo* or uncertain. Percentages (x axis) were computed after pooling counts across all father-proband or mother-proband duos. Variants that failed QC are excluded. **(B)** The false positive rate (fraction of non-*de novo* variants misclassified as *de novo*; y axis) of *duoNovo*, calculated separately for each father-proband and mother-proband duo. **(C)** The percentage of *de novo* variants detected from the entire trio (Methods) that received a classification from *duoNovo* (after QC filters) and were labeled as *de novo*, present on the haplotype inherited from the non-sequenced parent, or uncertain. Percentages (x axis) were computed after pooling counts across all father-proband or mother-proband duos. Variants that failed QC are excluded. **(D)** The percentage of trio-*de novo* variants classified by *duoNovo* as *de novo* (either in the father-proband or in the mother-proband duo), non-*de novo* (from both the father-proband and the mother-proband duo), or did not receive a classification. Each percentage is calculated separately for each trio.

To directly quantify the genome-scale false positive rate of *duoNovo*, we subsequently restricted our attention to candidate variants which we inferred – based on the full trio information – to have been transmitted from the non-sequenced parent (reliable heterozygous phased variant call in the proband and reliable heterozygous or homozygous variant call in the non-sequenced parent; reliable homozygous reference call in the sequenced parent). We examined the fraction of these variants that are classified as *de novo* (false positive rate; equivalent to the specificity), and found that *duoNovo* has an average false positive rate across father-proband and mother-proband duos equal to 1.2e-6, and 1.1e-6, respectively (Figure 3A, B). The majority (close to 60%) of these variants were correctly classified as present on the haplotype inherited from the non-sequenced parent, with slightly more than 40% labeled as uncertain (Figure 3A). In 23 of the 40 father-proband duos, and 24 of the 40 mother-proband duos, there were zero false positives (Figure 3B); the maximum false positive rate across all duos was 6.4e-6. As noted in the previous section, this is a conservative assessment since some of these variants may in fact be *de novo* even though they are present in the non-sequenced parent.

As an orthogonal assessment of the false positive rate, we applied *duoNovo* to swapped duos consisting of the father and the mother from each trio (that is, the proband was swapped with the parent previously treated as the non-sequenced one). From each swapped duo, we calculated the percentage of candidate variants that were classified as “*de novo*”, or as “present on the non-sequenced parent haplotype”. Either classification requires the detection of a haplotype block shared identical-by-descent between the two members of the duo. We would thus expect the percentage of classified variants from these swapped duos to be very low, since fathers and mothers share a much smaller percentage of their genome identical-by-descent compared to father-proband or mother-proband pairs. Indeed, we found that the median percentage across swapped duos is 0.03%, compared to 65% across true parent-proband duos (Supplemental Figure S6). One swapped duo stands out as an outlier, with 10.6% of variants receiving a classification (Supplemental Figure S6); this is substantially higher than all the rest, though still well below the percentage from true parent-proband duos (Supplemental Figure S6). Upon closer examination, we found that the father and mother from that swapped duo had the highest relatedness score among all father/mother pairs (Methods; Supplemental Figure S6), which could explain why *duoNovo* detected a larger fraction of identical-by-descent haplotype blocks.

### *duoNovo*’s sensitivity reflects known parent-of-origin effect

Finally, we sought to characterize the sensitivity of *duoNovo*, based on a set of *de novo* variants detected using the entire trio (hereafter referred to as trio-*de novo* variants; Methods), which we treated as the ground truth. Consistent with the aforementioned fact that most *de novo* variants are contributed by the fathers, we found that *duoNovo* collectively classified ~ 54% of trio-*de novo* variants as *de novo* from the father-proband duos, and only ~ 13% as *de novo* from the mother-proband duos (Figure 3C). Conversely, approximately 13% were classified as present on the maternally inherited haplotype from the father-proband duos, whereas about 47% were classified as present on the paternally inherited haplotype from the mother-proband duos (Figure 3C). 33% and 40% of variants were labeled as uncertain from the father-proband and mother-proband duos, respectively. Looking at the total fraction of trio-*de novo* variants that are classified as *de novo* (either from the father-proband or from the mother-proband duo; sensitivity), we found that the average sensitivity across all trios is 57% (Figure 3D). The remaining were all variants that were either labeled as uncertain or did not pass our QC filters; none of the trio-*de novo* variants were misclassified as non-*de novo* (Figure 3D).

### Variants classified as *de novo* fall into expected mutation subtypes

In agreement with previously reported mutational patterns among *de novo* variants (Kessler et al., 2020; Shojaeisaadi et al., 2024), we found that the single nucleotide variants *duoNovo* classified as *de novo* are mostly C>T and T>C substitutions (Supplemental Figure S4), with the C>T substitutions occurring both within and outside the CpG context. There was no statistically significant difference in the proportion of mutation types among the *de novo* classifications from father-proband duos (915 single nucleotide variants), and those from mother-proband duos (236 single nucleotide variants) (Supplemental Figure S4; chi-squared *p* = 0.4). We also found no statistically significant difference in the proportion of indels classified as *de novo* from father-proband versus mother-proband duos (Fisher’s exact test, *p* = 0.39).

## Discussion

We have developed and evaluated a method that enables the identification of *de novo* variants from parent-proband duos. Our approach is simple, and leverages the unique ability of LRS to both accurately detect and phase variants across large genomic segments. While it is established that LRS can detect variants that are undetectable with SRS, *duoNovo* illustrates that LRS can also enable the interpretation of variants that can be detected with SRS, but are hard-to-interpret.

Conceptually, *duoNovo* is based on the recognition that phasing can provide information about haplotype transmission, and thus reveal variant inheritance, without requiring access to both biological parent genotypes (see also Steyaert et al. (2024)). As such, it has the potential to improve variant interpretation in cases where sequencing both parents is not feasible, addressing a critical unmet need in clinical genetic testing. An inherent limitation of our approach is that it can only definitively classify *de novo* variants derived from the germline of the sequenced available parent. Given that the paternal germline contributes approximately 4 times as many *de novo* single nucleotide variants than the maternal germline (Jónsson et al., 2017; Kong et al., 2012; Sasani et al., 2019), this will limit the yield in assigning *de novo* status to variants identified clinically. However, regardless of whether it is applied to a father-proband or mother-proband duo, *duoNovo* can aid in the classification of clinically identified variants whose interpretation was otherwise limited by the unavailability of biologic family members.

While we have focused on single nucleotide variants and indels here, we anticipate that *duoNovo* is going to be similarly useful for additional variant types which LRS is capable of reliably detecting, such as structural variants and short tandem repeats (Beyter et al., 2021; Chaisson et al., 2015; Figueroa et al., 2024; Logsdon et al., 2020; Sedlazeck et al., 2018; Smolka et al., 2024). Finally, although we used PacBio HiFi LRS in this study, we note that *duoNovo* is generally applicable to any sequencing platform that generates accurate variant calls and enables read-backed haplotype reconstruction.

In summary, *duoNovo* is the first systematic method that can identify *de novo* variants from duos with high accuracy at the genome scale. It has the potential to increase the diagnostic yield of genetic testing for millions of families with diverse family structures, and may represent an example where long-read sequencing provides a clear benefit compared to short-read sequencing in the clinical diagnostic setting. We anticipate that its implementation and free availability as an R package will facilitate its use and adoption by the community.

## Methods

### Participants

We sequenced 122 individuals, from 38 trios and two quad families. Each of the two quad families was split into two trios, thus yielding a total of 42 trios. Two of these trios were then excluded from the analysis because more than 75% of candidate variants detected from the mother-proband duo failed QC due to low sequencing depth. Thus, all analyses were conducted with the remaining 40 trios. All individuals consented to provide samples for genetic testing to the Pediatrics Mendelian Genomics Research Center (part of the GREGoR consortium). The study was approved by Children’s National Hospital IRB (IRB #PRO00015852). Age (at childbirth) and genetic ancestry of the parents are provided in Supplemental Table 1.

### Long-read sequencing

Samples were prepared for LRS following the standard operating procedure (SOP) available at (PACB.com. (”Preparing whole genome and metagenome libraries using SMRTbell prep kit 3.0”). Sequencing was performed on the PacBio Revio system with the Revio polymerase Kit, following the Revio SMRT Link setup. Genomic DNA quality and concentration were assessed using the FEMTO Pulse (Agilent Technologies) and Qubit dsDNA HS reagents Assay kit (Thermo Fisher Scientific). SMRTbell libraries were constructed on the Hamilton Microlab Star and VENUS 5 system following the SOP available on PACB.com under ”Preparing whole genome and metagenome libraries using SMRTbell prep kit 3.0”. For library characterization, post size-selected SMRTbell Library samples were quantified using the Qubit DNA HS assay and DNA size was estimated using the FEMTO Pulse. The libraries were loaded at an On-Plate Concentration of 90 pM.

Raw sequencing reads were subsequently processed as follows. Circular consensus sequences were generated from subreads using CCS (version 7.0.0) with default settings, yielding demultiplexed HiFi reads. These reads were then aligned to the hg38 reference genome (https://github.com/PacificBiosciences/reference_genomes/blob/main/reference_genomes/human_GRCh38_no_alt_analysis_set/README.md) using PBMM2 (version 1.10.0), with default parameters.

### Variant calling

Variant calling following LRS was performed using DeepVariant (version 1.5.0; (Poplin et al., 2018; Yun et al., 2021)). For each duo as well as for each trio, gvcfs were first generated and joint variant calls were subsequently produced for each duo and trio separately with GLnexus (version 1.4.1) with the preset “DeepVariant unfiltered”. All our analyses and numbers reported are based on candidate *de novo* variants that were called both from a duo and its corresponding trio and thus were candidates we could evaluate.

For the evaluation of *duoNovo*’s performance, genotype calls from the non-sequenced parent at positions with PHRED genotype quality (GQ) at least 40 were used. Specifically, *de novo* classifications were determined to be correct if the non-sequenced parent did not have the candidate variant allele at that position (that is, if the non-sequenced parent was either homozygous for a non-variant allele or heterozygous with two different non-variant alleles). Similarly, non-*de novo* classifications were determined to be correct if the non-sequenced parent had the candidate variant allele at that position.

To obtain *de novo* variants from the entire trio, we first identified positions with a heterozygous variant in the proband and a homozygous reference call in both parents. Out of these positions, we then retained those with a minimum GQ of 40 for each sample, and a minimum depth of 20 reads for each sample. Multi-allelic variant sites were excluded. From the resulting set of *de novo* variants, we excluded variants clustered within the same phasing haplotype block, reasoning that these are likely to represent artifacts (see also below).

### Phasing

Following LRS, phasing was conducted using HiPhase (version 1.4.0) (Holt et al., 2024) with default parameters on the jointly called vcf from each duo. HiPhase assigned each phased variant to a phasing set. Variants in the same phasing set had the same phase relative to one another, enabling the resolution of those variants into haplotypes.

### Estimation of haplotype similarity and classification of candidate *de novo* variants with *duoNovo*

Following phasing, we imported the vcf files containing the phased variant calls into R with the VariantAnnotation package (Obenchain et al., 2014). We then only retained variant positions where sequencing depth was at least 20. With respect to GQ, we initially retained all positions with GQ at least 30. After obtaining classifications based on parent-proband sequence similarities computed using these positions (see next paragraph for details), we only retained classified variants with GQ at least 40 to ensure our results are not affected by genotyping errors. As stated in the Results, we defined candidate *de novo* variants as variants heterozygous in the proband and absent in the parent.

We then developed *duoNovo* to obtain classifications for these candidate variants. *duoNovo* first defines haplotype blocks as genomic regions within which: a) proband variants are assigned to the same phasing set; and b) parent variants are assigned to the same phasing set. *duoNovo* then evaluates candidate variants, separately in each of the two phasing orientations. It starts by discarding candidate variants that are either in haplotype blocks smaller than 10 kb, or at the boundaries of the remaining blocks (2 kb from the start/end coordinates). These boundary variants are discarded because we found that when they are classified as *de novo*, they tend to be false positives, especially in mother-proband duos (see below). Subsequently, *duoNovo* sequentially examines each of the proband haplotype blocks containing candidate *de novo* variants. Each of these haplotype blocks is compared to each of the two corresponding parental haplotype blocks using the Hamming distance. In addition, *duoNovo* performs the same comparisons for the other proband haplotype (not containing the candidate variant). In all cases, the Hamming distance between a pair of haplotype blocks is calculated after representing each haplotype block as a binary string (with 0 for the reference allele and 1 for the variant allele) and excluding candidate variants in the same phasing orientation as the candidate variant being evaluated. After performing these comparisons, *duoNovo* produces classifications based on the following criteria:

- If the proband haplotype block containing the candidate variant has a Hamming distance of 0 with only one of the two parental haplotype blocks, while the other proband haplotype block has a Hamming distance greater than 40 with both parental haplotype blocks, the candidate variant is classified as *de novo* (see below for an examination of the impact of the specific Hamming distance threshold).
- If the proband haplotype block containing the candidate variant has a Hamming distance greater than 40 with both parental haplotype blocks, while the other proband haplotype block has a Hamming distance of 0 with only one of the two parental haplotype blocks, the candidate variant is classified as inherited from the other parent.
- If none of the two above conditions were satisfied (e.g. due to no proband-parent haplotype pair having a Hamming distance of 0), the candidate variant does not receive a classification and is labeled as uncertain.

To minimize false positive classifications due to genotyping errors, from the resulting classifications we excluded variants falling within regions stratified as problematic by the Genome-in-a-Bottle consortium (Dwarshuis et al., 2024). Furthermore, as mentioned earlier, we labeled candidate variants with GQ less than 40 in either proband or parent as uncertain.

Finally, if two or more of the variants classified as *de novo* are present within the same haplotype block, *duoNovo* discards all *de novo* classifications in that block. While these could represent true *de novo* events, we have observed that they tend to be false positives (see section “Examining sources of false positive classifications”).

### Interpretation of Hamming distance equal to 0 between a proband-parent haplotype block pair

Intuitively, a Hamming distance equal to 0 between a proband-parent haplotype block pair indicates that this haplotype is shared identical-by-descent. This intuition can be made more precise when we consider the genotypes of the different positions that determine the Hamming distance.

1. **Positions where the proband is heterozygous and the parent is homozygous**. These positions provide direct information about haplotype transmission, because only one of the two proband haplotypes could have been transmitted from the sequenced parent. We note that, without read-backed phasing, candidate variants would always be classified as inherited from the non-sequenced parent, since they are heterozygous in the proband and absent in the sequenced parent. Hence, a candidate variant can only be classified as *de novo* using read-backed phasing. In turn, this implies that in order for the classification to be accurate, it is critical to ensure that the read-based phasing of variants in the region is accurate. We accomplish this using the two types of positions below.
2. **Positions where both proband and parent are heterozygous**. When considered in isolation, these positions do not provide direct information about haplotype transmission, because each of the two proband haplotypes could be identical-by-descent with the corresponding parental haplotype. However, when considered in the context of the haplotype inferred to have been transmitted from the sequenced parent based on 1 above, these positions serve as a quality control for the accuracy of the phasing in the region, in both the proband and the parent.
3. **Positions where the proband is homozygous and the parent is heterozygous**. Like 2, these do not provide direct information about haplotype transmission in isolation, because each of the two proband haplotypes could be identical-by-descent with the parental haplotype that harbors the allele for which the proband is homozygous. However, when considered in the context of the haplotype inferred to have been transmitted from the sequenced parent based on 1 above, they serve as a quality control for the accuracy of phasing in the parent, thus lending further support to the accuracy of phasing in the proband as described in 2.

Consequently, when the Hamming distance between a pair of proband-parent haplotype blocks is equal to 0, we can infer that: a) the proband inherited this haplotype block from the sequenced parent, and not from the other (non-sequenced) parent; b) the phasing of the candidate variant based on which we have determined whether the variant is *de novo* or not is accurate.

### Parameter sensitivity analysis

We examined the impact of *duoNovo*’s tuning parameters on the positive predictive value (PPV) and the number of *de novo* classifications, across all 80 duos.

First, we varied the threshold for the Hamming distance used to determine if a pair of proband-parent haplotype blocks is dissimilar and is thus not shared identical-by-descent (our default being 40). We found that a threshold of 0 always yields more *de novo* classifications compared to our default (as expected, since it is more lenient), but almost always yields a lower PPV, indicating that these classifications are enriched for false positives (Supplemental Figure S7, S8). Generally, we observed slight improvement when increasing thresholds up to 40, but no net gain when using thresholds above 40.

Second, we varied the distance threshold from the boundaries of haplotype blocks. By default, *duoNovo* excludes variants within 2kb of the start/end coordinates of haplotype blocks. We found that using a more lenient threshold tends to yield more false positives, especially in mother-proband duos (potentially reflecting a drop in phasing accuracy at these boundaries; Supplemental Figure S9). The impact on the number of *de novo* classifications is minimal, as expected given that this threshold affects the inclusion of a comparatively small number of variants (Supplemental Figure S10).

We then varied the thresholds for GQ and sequencing depth. One would naturally expect that using more lenient thresholds for candidate variants themselves would lead to an inflation of false positives false positives. However, the impact of these thresholds when focusing on the positions surrounding candidate variants – which determine the Hamming distance between each proband-parent haplotype block pair – is less obvious. We found that, while using more lenient GQ thresholds (10 or 20 instead of 30) appears to increase the PPV (Supplemental Figure S11), this is because a much smaller number of variants get classified as *de novo*. This is likely due to the fact that a lower GQ causes haplotype block pairs that in reality are identical-by-descent to not have a Hamming distance of 0 due to sequencing errors, thus leading to missed *de novo* variants. Finally, a more lenient sequencing depth threshold (10 instead of 20) had an overall minimal impact (Supplemental Figure S12).

### Examining sources of false positive classifications

As described above, from the resulting classifications *duoNovo* excludes variants clustered in the same haplotype block, and variants within regions annotated as problematic by the Genome-in-a-Bottle consortium (Dwarshuis et al., 2024). This is because we found that including these variants leads to an increased rate of false positive *de novo* classifications. Specifically, after pooling counts across all father-proband duos, we found that including variants within Genome-in-a-Bottle problematic regions reduced the precision from 98% to 90% in father-proband duoos, and from 91% to 71% in mother-proband duos. Moreover, including variants classified as *de novo* that are clustered in the same haplotype block reduced the precision to 70% in father-proband duos, and to 46% in mother-proband duos.

We also saw that, when only restricting to variants within genes (exonic and intronic regions either in coding or non-coding genes), there was no appreciable difference in the precision (97% in father-proband duos; 88% in mother-proband duos).

### Variant Annotation

We annotated all variants using ANNOVAR (Wang et al., 2010). For each variant, annotations included genomic compartments (e.g. exonic, intronic, intergenic), CpG vs non-CpG context, gnomAD version 4.1 allele frequencies, and whether the variant falls into a GIAB-problematic region (Dwarshuis et al., 2024).

### Relatedness and genetic ancestry calculation

Across all pairs of samples, relatedness was calculated using the “relate” function from Somalier (Pedersen et al., 2020) with default parameters. Genetic ancestry for each sample was predicted using the Somalier “ancestry” function using labeled data from the 1000 genomes project.

### Mutation types

We calculated the number of occurrences of the different types of single nucleotide variants and indels per duo with the MutationalPatterns R package (Manders et al., 2022). For Supplemental Figure S4, percentages were calculated after pooling the counts across all father-proband duos, and all mother-proband duos.

## Supplementary Material

Supplement 1

1

## Data Availability

Sequencing data are available on AnVIL as part of the GREGoR Consortium data release (https://gregorconsortium.org/data). *duoNovo* is freely available for installation as an R package at https://github.com/sbergercnmc/duonovo.
