# Supplementary material for: Identification of de novo variants from parent-proband duos via long-read sequencing
Source: medRxiv. 2025 Feb 26:2025.02.24.25322424. Preprint. [Version 1] doi: 10.1101/2025.02.24.25322424 (PMC11888490; doi:10.1101/2025.02.24.25322424)
Supplement: 1 [file NIHPP2025.02.24.25322424V1-supplement-1.pdf]

medRxiv preprint doi: https://doi.org/10.1101/2025.02.24.25322424; this version posted February 26, 2025. The copyright holder has placed this preprint (which was not certified by peer review) in the Public Domain. It is no longer restricted by copyright. Anyone can legally share, reuse, remix, or adapt this material for any purpose without crediting the original authors.

Supplemental Figures

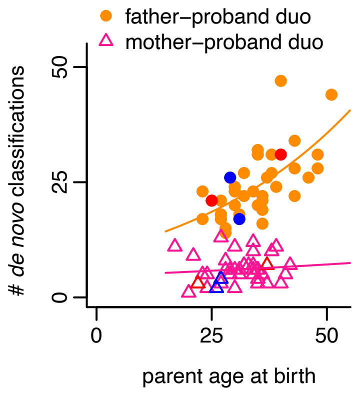

Supplemental Figure S1. Paternal age positively correlates with the number of *de novo* classifications from father-proband duos. Each point corresponds to a duo. Trend lines were plotted after fitting Poisson regression models using the log link. The red and blue points correspond to 2 father-proband and 2 mother-proband duos that share the same father and mother, respectively (from the 2 quad families that were each split into 2 trios).

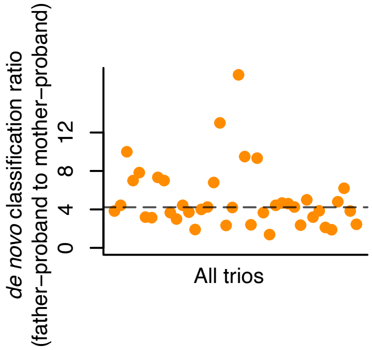

Supplemental Figure S2. *duoNovo* detects more *de novo* variants from father-proband duos compared to mother-proband duos. Each point corresponds to a trio and its position on the y axis corresponds to the ratio of the number of *de novo* classifications from the father-proband duo to the number of *de novo* classifications from the mother-proband duo. The dashed horizontal line corresponds to the median across all trios.

medRxiv preprint doi: https://doi.org/10.1101/2025.02.24.25322424; this version posted February 26, 2025. The copyright holder has placed this preprint (which was not certified by peer review) in the Public Domain. It is no longer restricted by copyright. Anyone can legally share, reuse, remix, or adapt this material for any purpose without crediting the original authors.

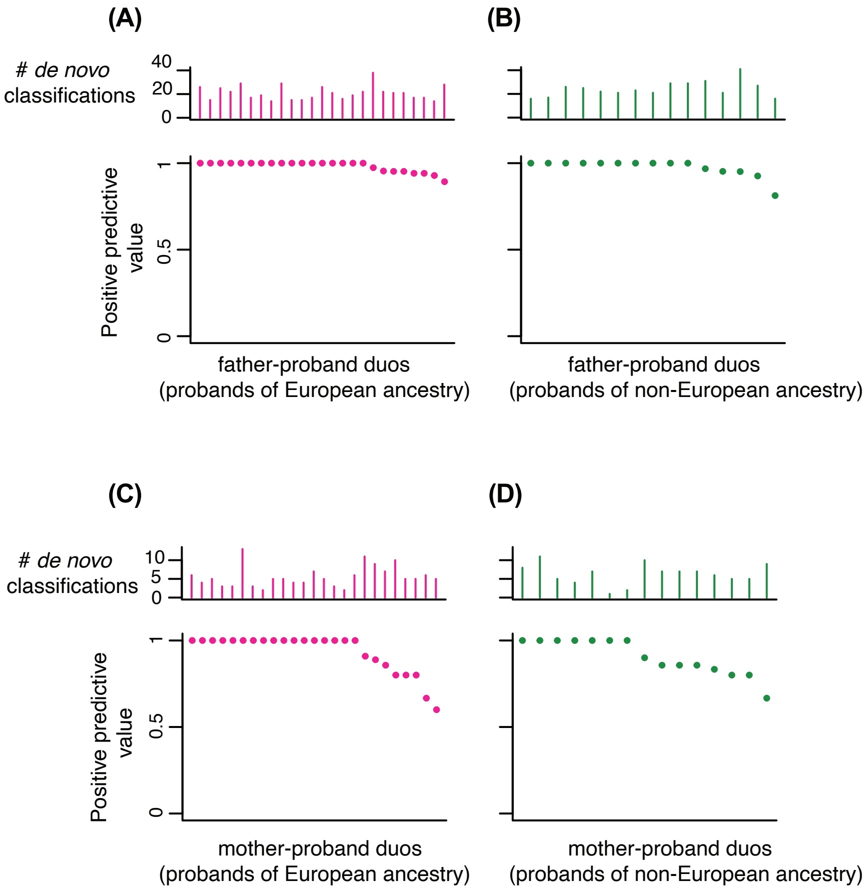

Supplemental Figure S3. No difference in the positive predictive value of *duoNovo* in probands of European vs non-European ancestry. Like Figure 2A, C, but shown separately for duos with probands of European versus non-European ancestry. Ancestry was calculated using Somalier (Pedersen et al., 2020). Non-European ancestry groups include African (2 probands), Admixed American (7 probands), East Asian (3 probands), and South Asian (3 probands).

medRxiv preprint doi: https://doi.org/10.1101/2025.02.24.25322424; this version posted February 26, 2025. The copyright holder has placed this preprint (which was not certified by peer review) in the Public Domain. It is no longer restricted by copyright. Anyone can legally share, reuse, remix, or adapt this material for any purpose without crediting the original authors.

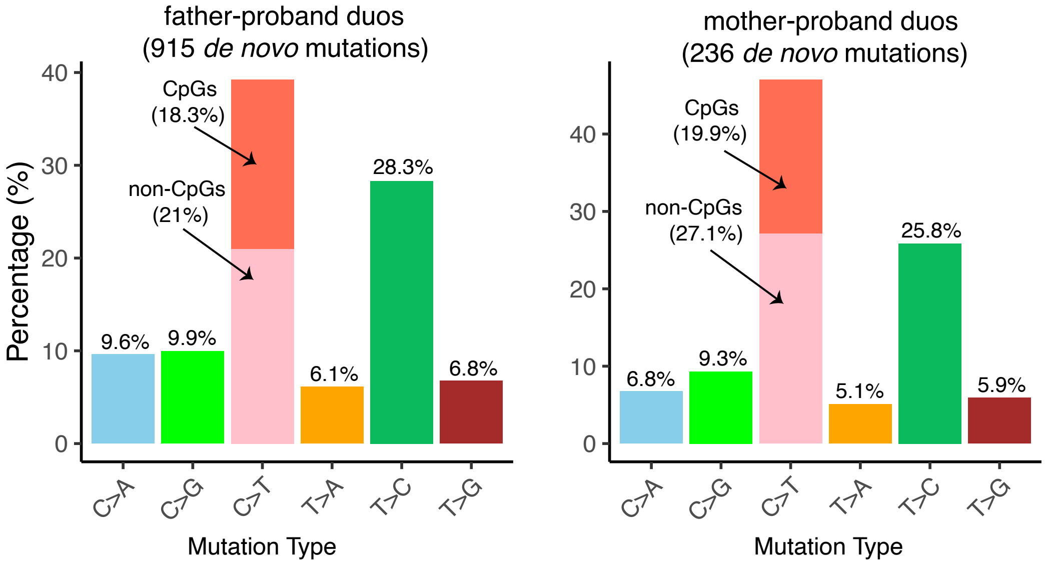

Supplemental Figure S4. The *de novo* single nucleotide variants identified by *duoNovo* fall into expected mutation subtypes. Percentages were computed after pooling all single nucleotide variants classified as *de novo* from the father-proband duos (left) and the mother-proband duos (right).

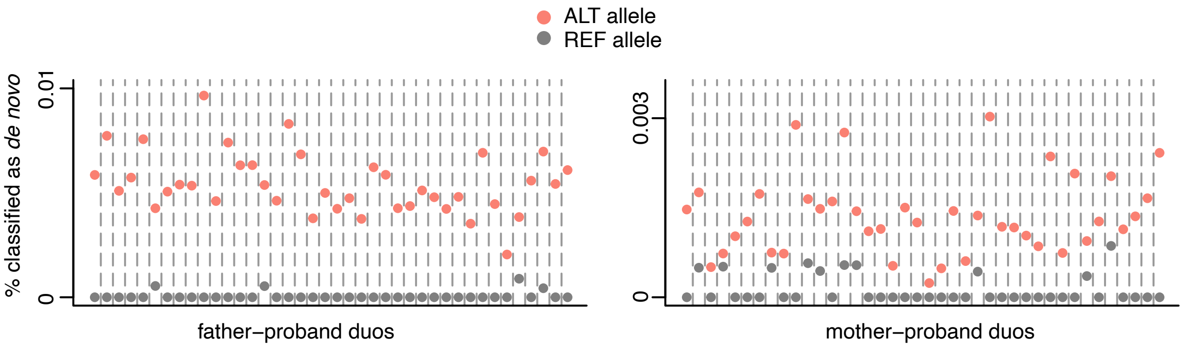

Supplemental Figure S5. *duoNovo* very rarely classifies candidate reference alleles as *de novo*. Candidate alternative (ALT) alleles were identified by finding positions where the proband had the "1|0" or "0|1" genotype and the parent had the "0/0" genotype. Conversely, candidate reference (REF) alleles were identified by finding positions where the proband had the "1|0" or "0|1" genotype and the parent had the "1/1" genotype. Positions were either the proband or the parent failed QC filters (Methods) were excluded.

medRxiv preprint doi: https://doi.org/10.1101/2025.02.24.25322424; this version posted February 26, 2025. The copyright holder has placed this preprint (which was not certified by peer review) in the Public Domain. It is no longer restricted by copyright. Anyone can legally share, reuse, remix, or adapt this material for any purpose without crediting the original authors.

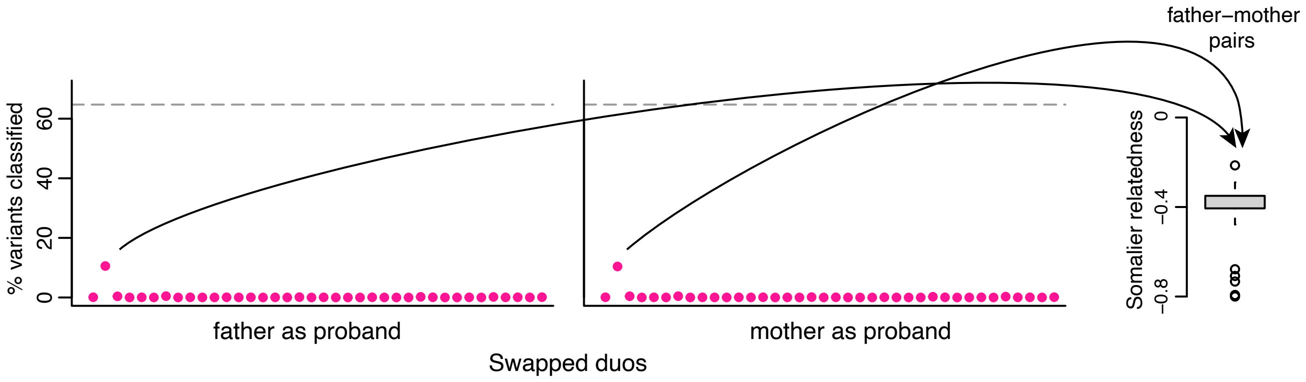

Supplemental Figure S6. *duoNovo* does not classify variants from swapped duos. Each point corresponds to a swapped duo, and its position on the y axis indicates the percentage of variants that received a classification (either *de novo* or on the non-sequenced parent haplotype). Each swapped duo consists of the two parents instead of a parent and the proband; *duoNovo* was applied to each swapped duo by either treating the father as the proband (left) or the mother as the proband (right). The dashed horizontal line corresponds to the median percentage of variants that received a classification across all regular (father-proband or mother-proband) duos. The rightmost panel depicts the distribution of relatedness coefficients between all the fathers and mothers (each point corresponds to a father-mother pair), showing that the outlier swapped duo is the one with the highest relatedness coefficient.

medRxiv preprint doi: https://doi.org/10.1101/2025.02.24.25322424; this version posted February 26, 2025. The copyright holder has placed this preprint (which was not certified by peer review) in the Public Domain. It is no longer restricted by copyright. Anyone can legally share, reuse, remix, or adapt this material for any purpose without crediting the original authors.

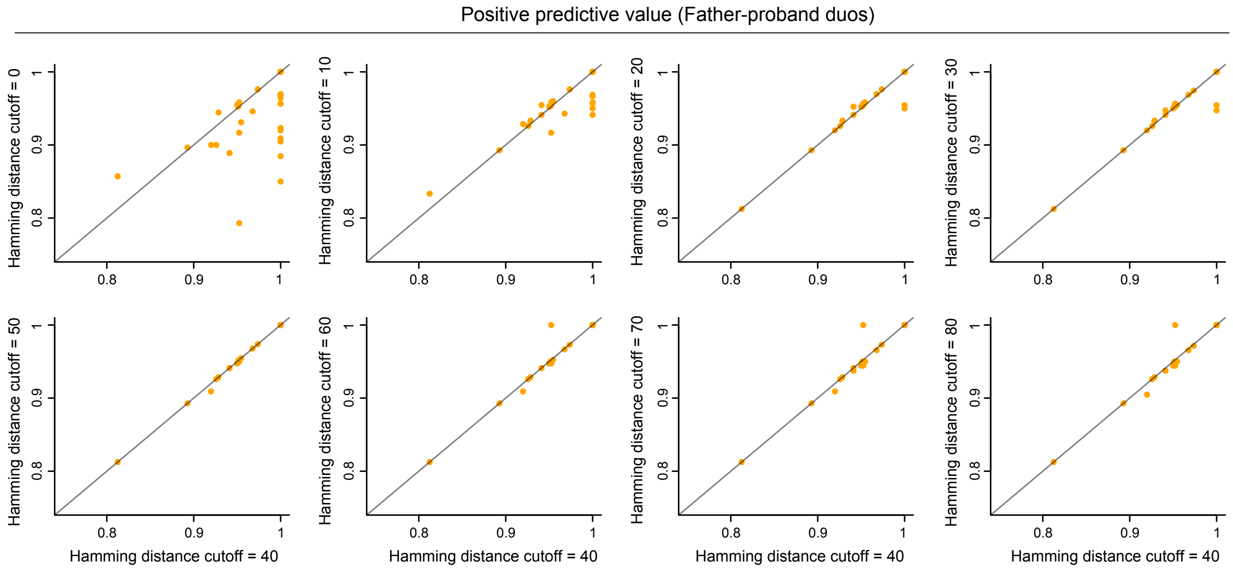

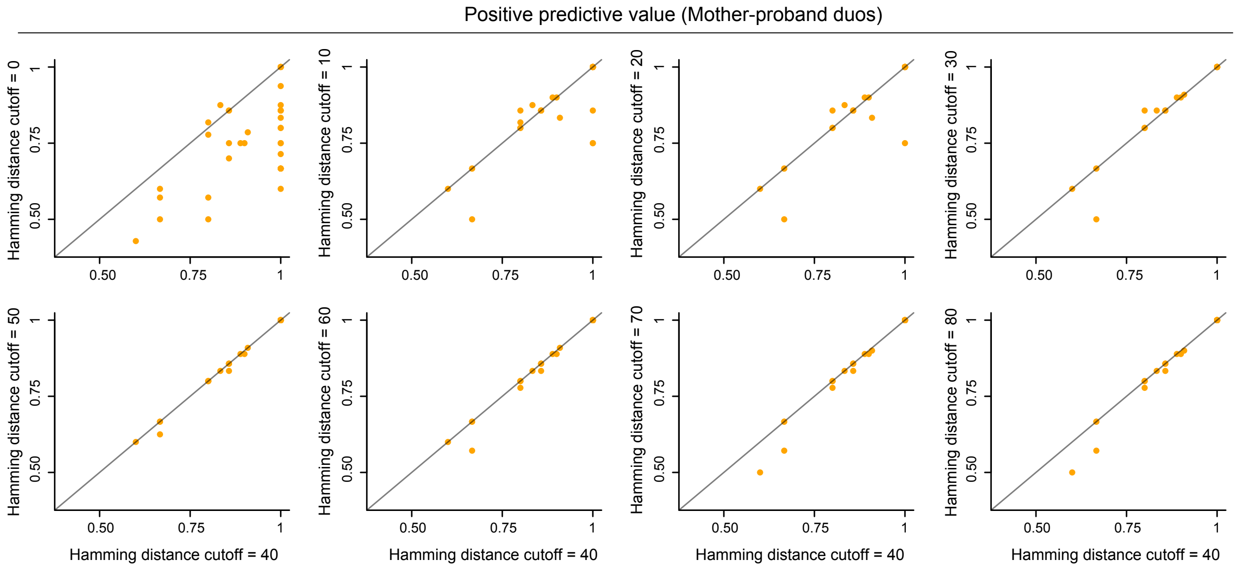

Supplemental Figure S7. The impact of the Hamming distance threshold for determining dissimilarity between a pair of haplotype blocks on the positive predictive value. Each point corresponds to a duo. The x-axis shows the positive predictive value (PPV) when using a Hamming distance threshold of 40 (default), and the y axis shows the PPV when using different Hamming distance thresholds. There is some degree of overplotting, due to duos that have the same or highly similar PPV.

medRxiv preprint doi: https://doi.org/10.1101/2025.02.24.25322424; this version posted February 26, 2025. The copyright holder has placed this preprint (which was not certified by peer review) in the Public Domain. It is no longer restricted by copyright. Anyone can legally share, reuse, remix, or adapt this material for any purpose without crediting the original authors.

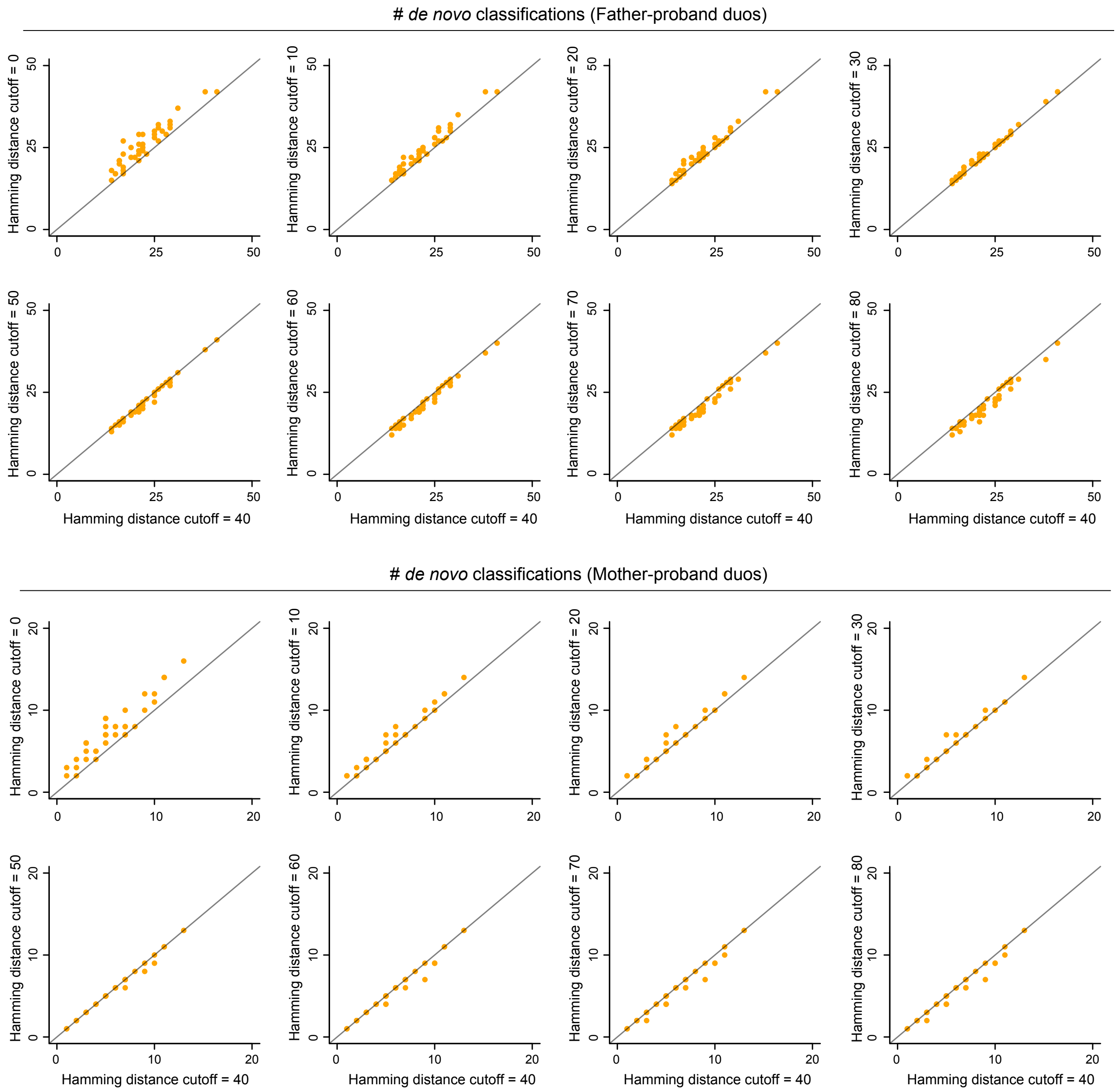

Supplemental Figure S8. The impact of the Hamming distance threshold for determining dissimilarity between a pair of haplotype blocks on the number of *de novo* classifications. Like Supplemental Figure S7, but depicting the number of *de novo* classifications instead of the PPV.

medRxiv preprint doi: https://doi.org/10.1101/2025.02.24.25322424; this version posted February 26, 2025. The copyright holder has placed this preprint (which was not certified by peer review) in the Public Domain. It is no longer restricted by copyright. Anyone can legally share, reuse, remix, or adapt this material for any purpose without crediting the original authors.

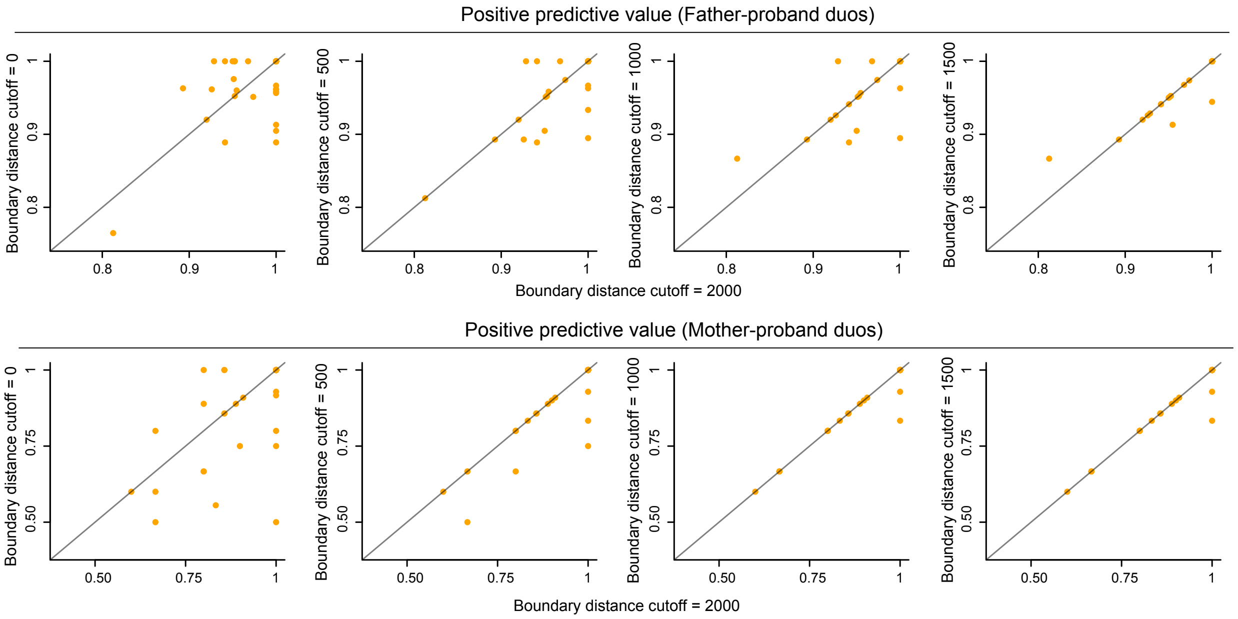

Supplemental Figure S9. The impact of the distance threshold from haplotype block boundaries on the positive predictive value. Like Supplemental Figure S7, but showing the impact of the threshold for the distance from the haplotype block boundaries (start/end coordinates) on the PPV.

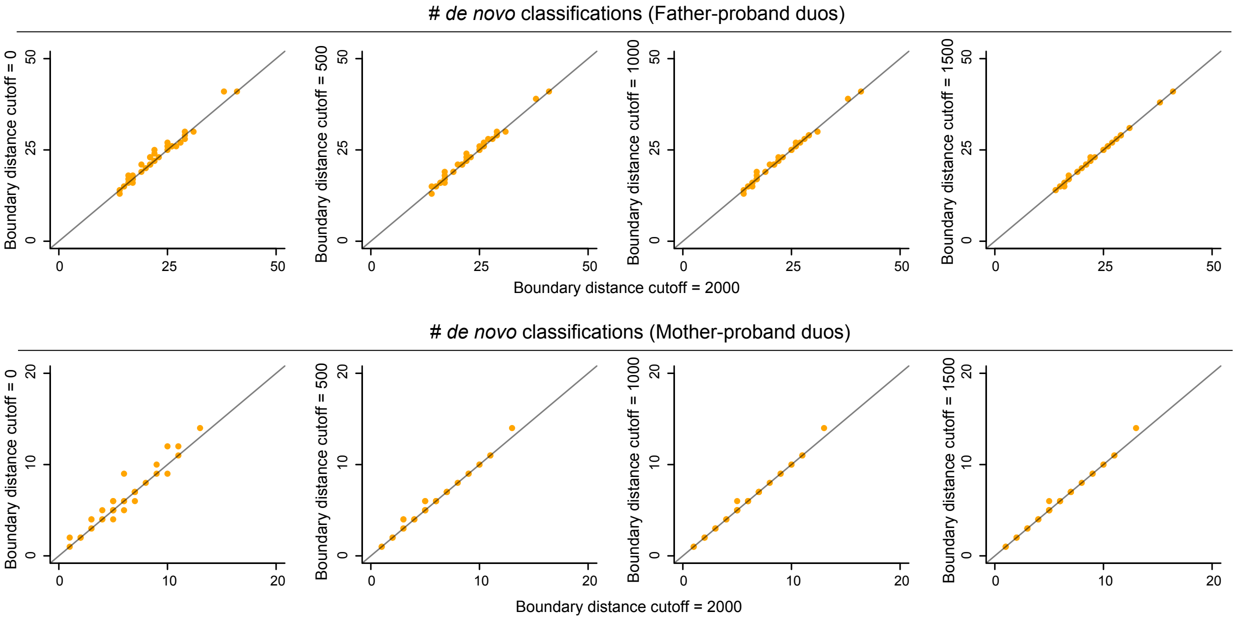

Supplemental Figure S10. The impact of the distance threshold from haplotype block boundaries on the number of *de novo* classifications. Like Supplemental Figure S8, but showing the impact of the threshold for the distance from the haplotype block boundaries (start/end coordinates) on the number of *de novo* classifications.

medRxiv preprint doi: https://doi.org/10.1101/2025.02.24.25322424; this version posted February 26, 2025. The copyright holder has placed this preprint (which was not certified by peer review) in the Public Domain. It is no longer restricted by copyright. Anyone can legally share, reuse, remix, or adapt this material for any purpose without crediting the original authors.

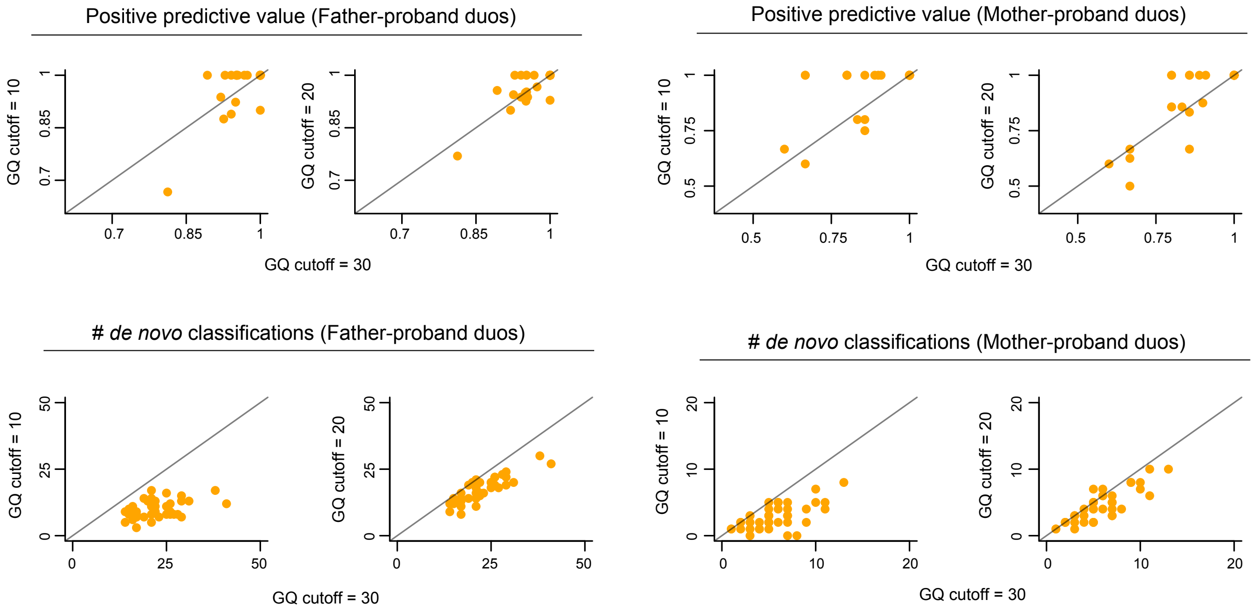

Supplemental Figure S11. The impact of the phred quality (GQ) threshold at positions surrounding candidate variants on the positive predictive value and the number of *de novo* classifications. Like Supplemental Figure S7 and Supplemental Figure S8, but showing the impact of the GQ threshold for positions surrounding the candidate variant (that is, positions which determine the Hamming distance) on the PPV and the number of *de novo* classifications.

medRxiv preprint doi: https://doi.org/10.1101/2025.02.24.25322424; this version posted February 26, 2025. The copyright holder has placed this preprint (which was not certified by peer review) in the Public Domain. It is no longer restricted by copyright. Anyone can legally share, reuse, remix, or adapt this material for any purpose without crediting the original authors.

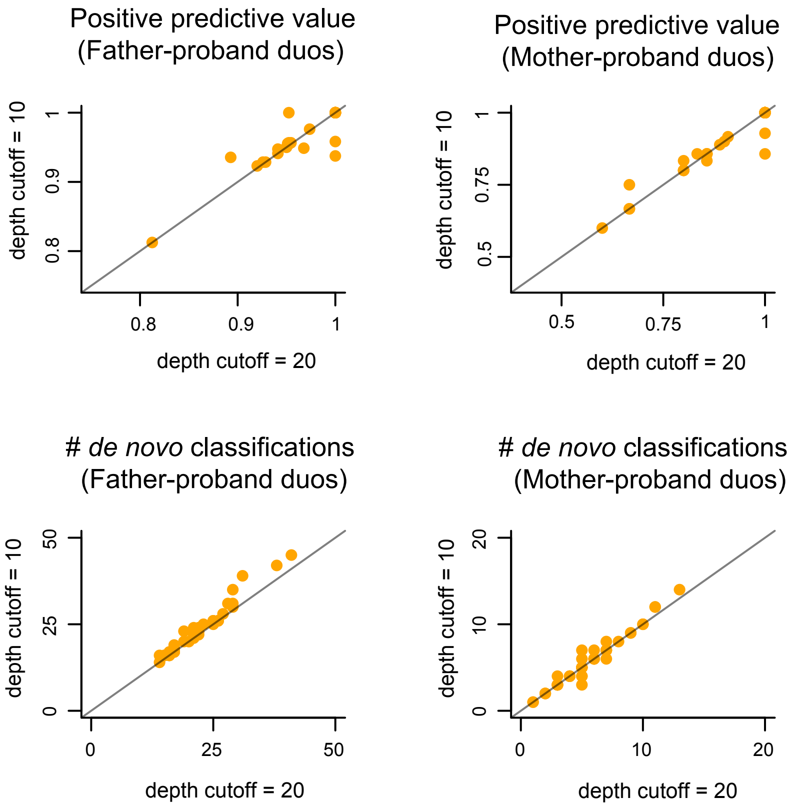

Supplemental Figure S12. The impact of the sequencing depth threshold at positions surrounding candidate variants on the positive predictive value and the number of *de novo* classifications. Like Supplemental Figure S11, but showing the impact of the sequencing depth threshold for positions surrounding the candidate variant (that is, positions which determine the Hamming distance) on the PPV and the number of *de novo* classifications.
